# Predictive value of atherogenic index of plasma and atherogenic index of plasma combined with low-density lipoprotein cholesterol for the risk of acute myocardial infarction

**DOI:** 10.3389/fcvm.2023.1117362

**Published:** 2023-05-26

**Authors:** Min Chen, Cao-yang Fang, Jiong-chao Guo, La-mei Pang, Yuan Zhou, Yu Hong, Lin-fei Yang, Jing Zhang, Ting Zhang, Bing-feng Zhou, Guang-quan Hu

**Affiliations:** ^1^ Department of Cardiology, The Second People’s Hospital of Hefei, Hefei Hospital Affiliated to Anhui Medical University, Hefei, China; ^2^ Department of Cardiology, The Third Affiliated Hospital of Anhui Medical University (The First People’s Hospital of Hefei), Hefei, China; ^3^ Department of Endocrinology, Hefei BOE Hospital, Hefei, China; ^4^ Department of Cardiology, The Second Affiliated Hospital of Anhui Medical University, Hefei, China

**Keywords:** lipid metabolism, atherogenic index of plasma, LDL cholesterol, acute myocardial infarction, cardiovascular disease

## Abstract

**Background and aims:**

Acute myocardial infarction (AMI) is a prevalent medical condition associated with significant morbidity and mortality rates. The principal underlying factor leading to myocardial infarction is atherosclerosis, with dyslipidemia being a key risk factor. Nonetheless, relying solely on a single lipid level is insufficient for accurately predicting the onset and progression of AMI. The present investigation aims to assess established clinical indicators in China, to identify practical, precise, and effective tools for predicting AMI.

**Methods:**

The study enrolled 267 patients diagnosed with acute myocardial infarction as the experimental group, while the control group consisted of 73 hospitalized patients with normal coronary angiography. The investigators collected general clinical data and relevant laboratory test results and computed the Atherogenic Index of Plasma (AIP) for each participant. Using acute myocardial infarction status as the dependent variable and controlling for confounding factors such as smoking history, fasting plasma glucose (FPG), low-density lipoprotein cholesterol (LDL-C), blood pressure at admission, and diabetes history, the researchers conducted multivariate logistic regression analysis with AIP as an independent variable. Receiver operating characteristic (ROC) curves were employed to determine the predictive value of AIP and AIP combined with LDL-C for acute myocardial infarction.

**Result:**

The results of the multivariate logistic regression analysis indicated that the AIP was an independent predictor of acute myocardial infarction. The optimal cut-off value for AIP to predict AMI was −0.06142, with a sensitivity of 81.3%, a specificity of 65.8%, and an area under the curve (AUC) of 0.801 (95% confidence interval [CI]: 0.743–0.859, *P* < 0.001). When AIP was combined with LDL-C, the best cut-off value for predicting acute myocardial infarction was 0.756107, with a sensitivity of 79%, a specificity of 74%, and an AUC of 0.819 (95% CI: 0.759–0.879, *P* < 0.001).

**Conclusions:**

The AIP is considered an autonomous determinant of risk for AMI. Utilizing the AIP index alone, as well as in conjunction with LDL-C, can serve as effective predictors of AMI.

## 1. Introduction

The incidence of AMI constitutes a significant contributor to global mortality, ranking among the foremost causes thereof (1, 2). Atherogenesis and plaque formation in the subintimal coronary artery layers are influenced by lipid profiles and thus serve as predictive indicators of AMI (3–5). Previous research has indicated a significant association between LDL-C and atherosclerosis, making it the primary focus of lipid-lowering therapy (6). Additionally, LDL-C levels have been linked to pulse wave velocity, a predictor of cardiovascular events (7). Despite the effective control of LDL-C levels, the prevalence of atherosclerotic cardiovascular disease (ASCVD) remains high (8). The 2021 ESC Guidelines on cardiovascular disease prevention in clinical practice suggest Non-HDL Cholesterol as a reasonable alternative treatment goal for all patients, particularly for those with hypertriglyceridemia or diabetes mellitus (DM) (9). However, single changes in serum lipid levels cannot provide complete predictability of the occurrence and prognosis of AMI. Consequently, new comprehensive lipid indicators have become a recent research focal point.

A newly developed index, AIP, has been shown to indirectly reflect the particle size of small dense low-density lipoprotein cholesterol (sdLDL-C), which is more effective in predicting cardiovascular risk than traditional lipid parameters such as triglyceride (TG) and LDL-C (10). AIP can serve as a plasma marker of atherosclerosis and quantify abnormal lipid metabolism, enabling the assessment of the risk of atherosclerosis to a certain extent (11, 12). Thus, an analysis of the clinical data of patients with AMI was conducted to investigate the relationship between plasma arteriosclerosis index and AMI and provide a clinical basis for the active prevention and treatment of this disease.

## 2. Subjects and methods

### 2.1. Study subjects

This retrospective case study was conducted using data obtained from the hospital's electronic medical record system query system between December 2018 and March 2022. The diagnosis of AMI was confirmed through emergency coronary angiography (CAG) following symptoms of chest pain and chest tightness. The diagnosis of AMI following the 4th Universal Definition of Myocardial Infarction as established by the European Society of Cardiology/American College of Cardiology/American Heart Association/World Federation of Heart Disease (EACS/ACC/AHA/WHF) in 2018. Patients who met any of the following exclusion criteria were not included in the study: (1) history of previous AMI, coronary artery bypass grafting, or percutaneous coronary intervention (PCI). (2) previous heart transplantation. (3) incomplete medical history, incomplete clinical data, or absence of coronary angiography results. (4) concomitant other cardiac diseases requiring surgery (5) the presence of malignant tumors, severe liver or kidney diseases, hereditary hyperlipidemia, or congenital cardiovascular disease (CVD). (6) use of lipid-lowering drugs within the previous 3 months.

### 2.2. Methods

In this study, relevant information was extracted from the hospital's electronic medical record system. The extracted information included demographic data such as gender and age, as well as admission blood pressure, history of hypertension, diabetes, smoking, and drinking. In addition, laboratory tests were conducted to measure FPG, uric acid (UA), creatinine (Cr), total cholesterol (TC), TG, LDL-C, and high-density lipoprotein cholesterol (HDL-C).

### 2.3. Calculate the AIP

AIP was calculated according to the formula of AIP=log⁡(TG/HDL−C).

### 2.4. Statistic analysis

The statistical software SPSS version 26 was utilized to conduct data analysis. Descriptive statistics such as mean and standard deviation were used to express continuous variables. The normality of data in two groups was compared using independent sample t-test, while analysis of variance (ANOVA) was used for comparing data in multiple groups. Rank sum test was employed for comparing skewed data among two or more groups. Categorical variables were expressed as frequency or percentage (%), and the *χ*2 test was used for comparison between the groups. Binary logistic regression analysis was performed to evaluate the predictive value of AIP for acute myocardial infarction, and adjusted odds ratio (OR) value with a 95% CI was calculated. ROC curves were used to illustrate the diagnosis of acute myocardial infarction by AIP and AIP combined with LDL-C, and sensitivity and specificity were determined based on the maximum Youden index. All statistical analyses were conducted using two-tailed tests, with a significance level of *P *< 0.05.

## 3. Result

### 3.1. General clinical data of the two groups

**Figure 1:**
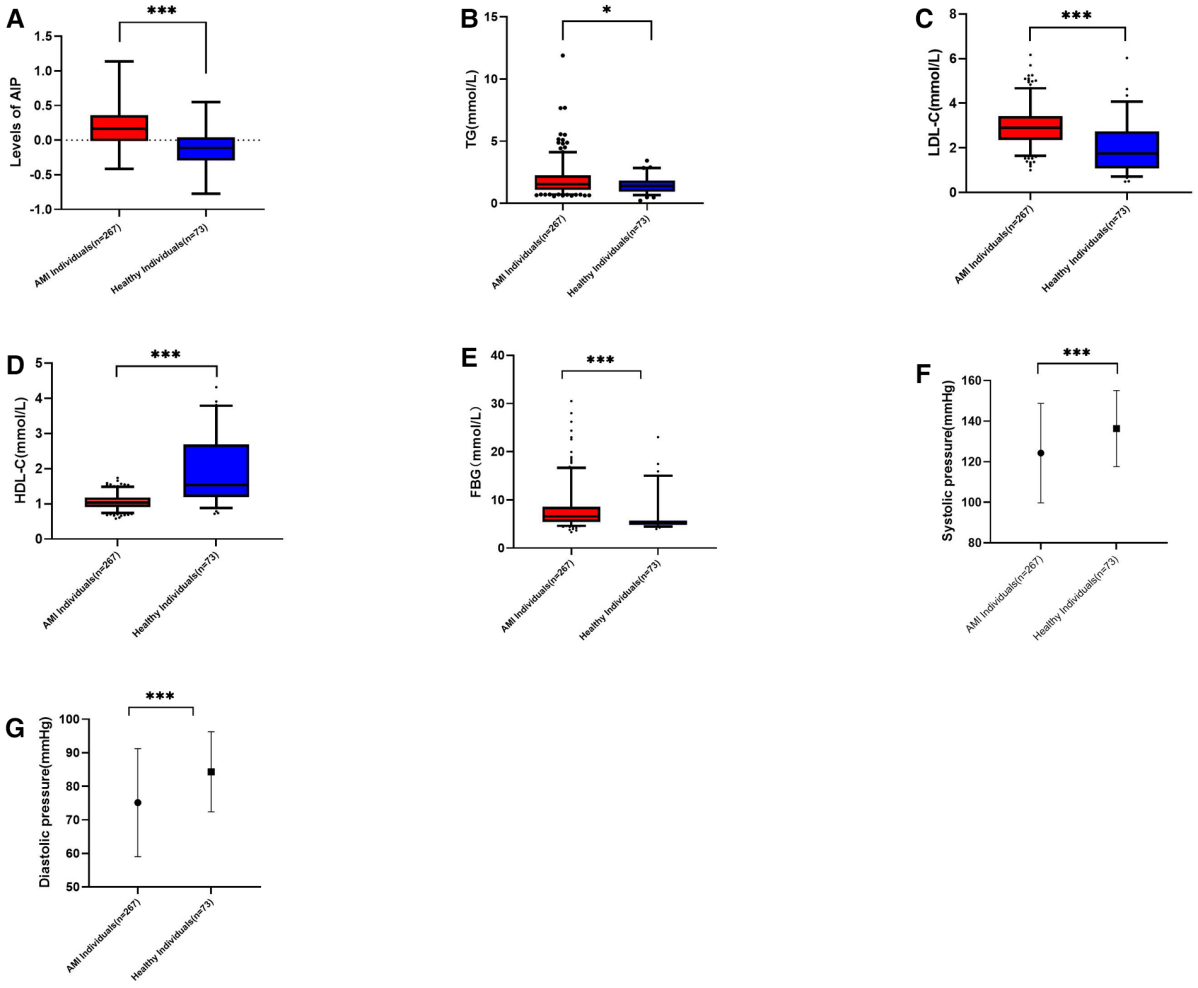
(**A**): AIP levels in myocardial infarction group and control group, *p* < 0.001. (**B**): TG in myocardial infarction group and control group, *p* = 0.02. (**C**): LDL-C in myocardial infarction group and control group, *p* < 0.001. (**D**): HDL-C in myocardial infarction group and control group, *p* < 0.001. (**E**): HDL-C myocardial infarction group and control group, *p* < 0.001. (**F**): Systolic pressure in myocardial infarction group and control group, *p* < 0.001. (**G**): Diastolic pressure in myocardial infarction group and control group, *p* < 0.001.

**Figure 2:**
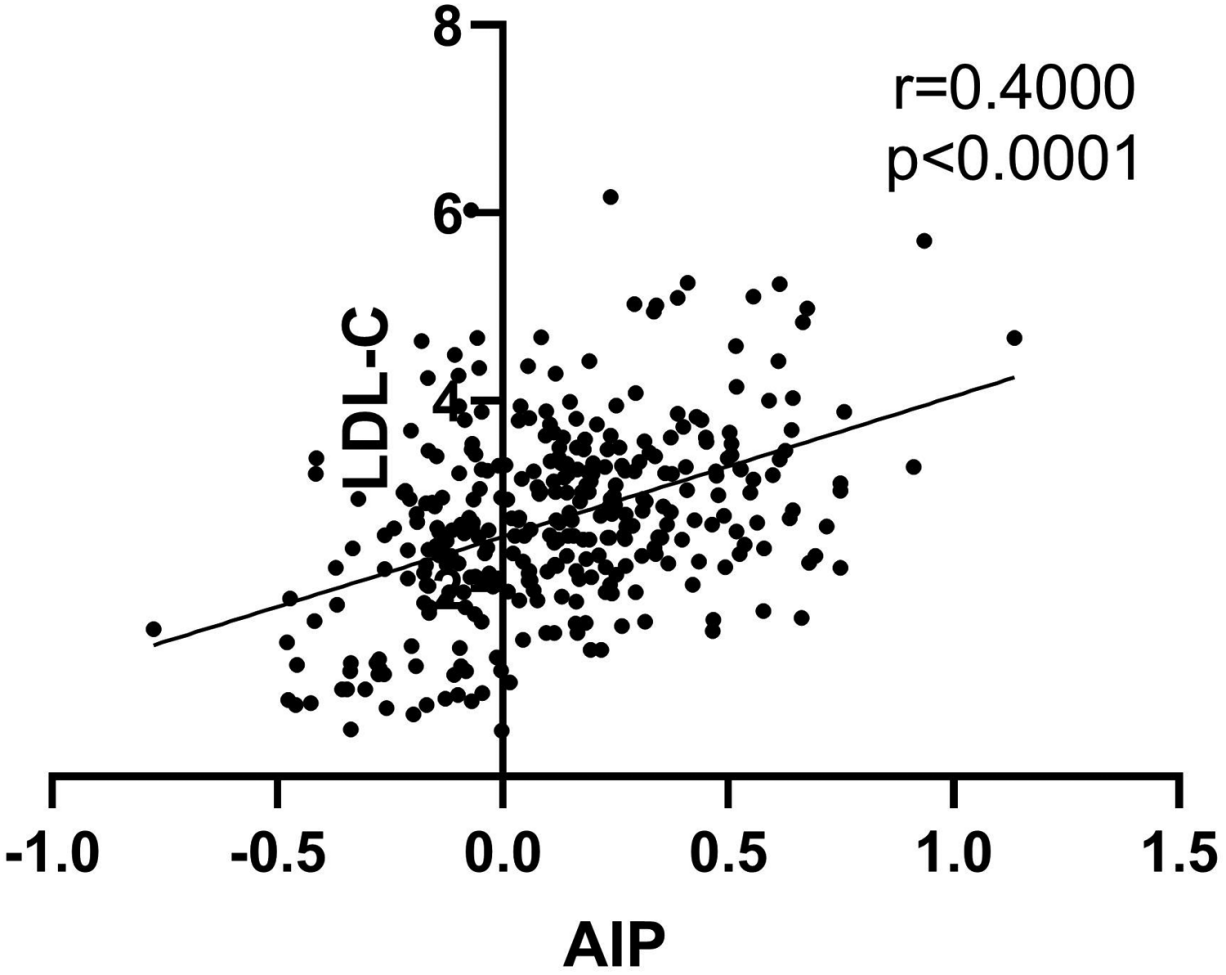
Correlation analysis of the AIP with the concentration of LDL-C.

Table 1 displays the baseline characteristics and biochemical indicators of the study population. The results indicate no significant differences in age (*P *= 0.148), gender (*P *= 0.838), drinking (*P *= 0.417), and history of hypertension (*P *= 0.866) between the two groups (*P *> 0.05). However, significant differences were observed in smoking (*P *< 0.001), history of diabetes (*P *= 0.002), FPG (*P *< 0.001), TG (*P *< 0.02), AIP (*P *< 0.001), HDL-C (*P *< 0.001), LDL-C (*P *< 0.001), systolic blood pressure, and diastolic blood pressure at admission (*P *< 0.001) between the two groups (*P *< 0.05) (Table 1) (Figure 1). Upon conducting a subgroup analysis focusing on myocardial infarction, significant differences were observed in variables such as systolic pressure, diastolic pressure, TC (mmol/L), HDL-C (mmol/L), LDL-C (mmol/L), and Cr (*P *< 0.05) (Table 1) (Figure 1). Additionally, we conducted a correlation analysis and found that AIP was significantly correlated with LDL-C (*r* = 0.4, *P* < 0.001) (Figure 2).

**Table 1:** Comparison of clinical data of patients [*n*(%), x ± s/M(P25,P75)].

Clinical data	AMI individuals (*n* = 267)	Non-AMI individuals (*n* = 73)	*P*	AMI individuals (*n* = 267)			
				LAD (*n* = 125)	LCX (*n* = 61)	RCA (*n* = 81)	*P*
Age (years)	60.33 ± 13.90	62.43 ± 9.97	0.148	59.37 ± 13.07	60.08 ± 15.57	61.99 ± 13.85	0.414
Male	215 (80.5%)	58 (79.5%)	0.838	106 (84.8%)	51 (83.6%)	58 (71.6%)	0.051
Smoking status	157 (58.8%)	18 (24.7%)	<0.001*	72 (57.6%)	40 (65.6%)	45 (55.6%)	0.453
Hypertension	147 (55.1%)	41 (56.2%)	0.866	64 (51.2%)	30 (49.2%)	53 (65.4%)	0.077
Diabetes	71 (26.6%)	7 (9.6%)	0.002*	31 (24.8%)	20 (33.3%)	20 (24.7%)	0.460
Systolic pressure	124.30 ± 24.50	136.37 ± 18.75	<0.001*	129.74 ± 23.6	123.66 ± 26.4	116.37 ± 22.4	0.001*
Diastolic pressure	75.13 ± 16.10	84.33 ± 11.95	<0.001*	78.75 ± 13.93	74.97 ± 20.73	69.67 ± 13.70	<0.001*
TG (mmol/L)	1.89 ± 1.28	1.46 ± 0.63	0.020*	1.82 ± 1.12	1.86 ± 1.07	2.03 ± 1.63	0.788
TC (mmol/L)	4.54 ± 1.02	4.36 ± 1.15	0.198	4.75 ± 0.99	4.78 ± 1.19	4.26 ± 0.84	0.003*
HDL-C (mmol/L)	1.06 ± 0.22	1.92 ± 0.94	<0.001*	1.09 ± 0.20	1.07 ± 0.27	1.01 ± 0.21	0.024*
LDL-C (mmol/L)	2.95 ± 0.86	1.99 ± 1.12	<0.001*	3.10 ± 0.90	2.94 ± 0.91	2.72 ± 0.70	0.005*
Cr	74.43 ± 34.75	71.89 ± 19.75	0.960	68.66 ± 20.15	76.98 ± 32.79	81.44 ± 49.73	0.026*
FPG	8.05 ± 4.22	6.03 ± 3.16	<0.001*	8.00 ± 4.24	8.91 ± 5.37	7.47 ± 2.97	0.331
AIP	0.19 ± 0.25	−0.11 ± 0.25	<0.001*	0.17 ± 0.25	0.19 ± 0.28	0.23 ± 0.26	0.245
Uric Acid	366.51 ± 106.85	381.07 ± 113.54	0.31	355.74 ± 102.19	369.05 ± 116.42	381.21 ± 105.84	0.243

(TG), triglyceride; (TC), total cholesterol; (HDL-C), high-density lipoprotein cholesterol; (LDL-C), low-density lipoprotein cholesterol; (Cr), creatinine; (FPG), fasting plasma glucose; (AIP), Atherogenic index of plasma; (LAD), left anterior descending; (LCX), left circumflex; (RCA), right coronary artery; (AMI), acute myocardial infarction.

* *P* < 0.05.

### 3.2. Multivariate logistic regression analysis

In this study, a multivariate logistic regression analysis was conducted to examine the relationship between AIP and the risk of AMI after adjusting for potential confounding variables, including smoking, history of diabetes, systolic blood pressure at admission, diastolic blood pressure at admission, FPG, and LDL-C. To avoid multicollinearity, TG and HDL-C were excluded from the regression model since AIP was calculated from these variables. The results revealed that AIP (OR=31.846, *P* < 0.001, 95% CI = 6.098–166.314), LDL-C (OR = 2.492, *P* < 0.001, 95% CI = 1.623–3.827), history of smoking (OR = 4.627, *P *< 0.001, 95% CI = 2.137–10.021), and FPG (OR = 1.181, *P *= 0.033, 95% CI = 1.014–1.376) were independent predictors of AMI after adjustment. Conversely, systolic blood pressure (OR = 0.985) and diastolic blood pressure (OR = 0.963) were found to be protective factors for AMI (Table 2).

**Table 2:** Multivariate logistic regression analysis.

Variable	OR	95% CI	*P*
AIP	31.846	6.098–166.314	<0.001*
LDL-C	2.492	1.623–3.827	<0.001*
Systolic pressure (mmHg)	0.985	0.964–1.007	0.179
Diastolic pressure (mmHg)	0.963	0.934–0.994	0.020*
Diabetes	1.788	0.525–6.084	0.352
Smoking	4.627	2.137–10.021	<0.001*
FPG	1.181	1.014–1.376	0.033*

(LDL-C), low-density lipoprotein cholesterol; (AIP), Atherogenic index of plasma; (FPG), fasting plasma glucose; (OR) value, odds ratio; (95% CI) 95% confidence interval; (ROC) curves, Receiver operating characteristic.

* *P* < 0.05.

### 3.3. ROC curve analysis

**Figure 3:**
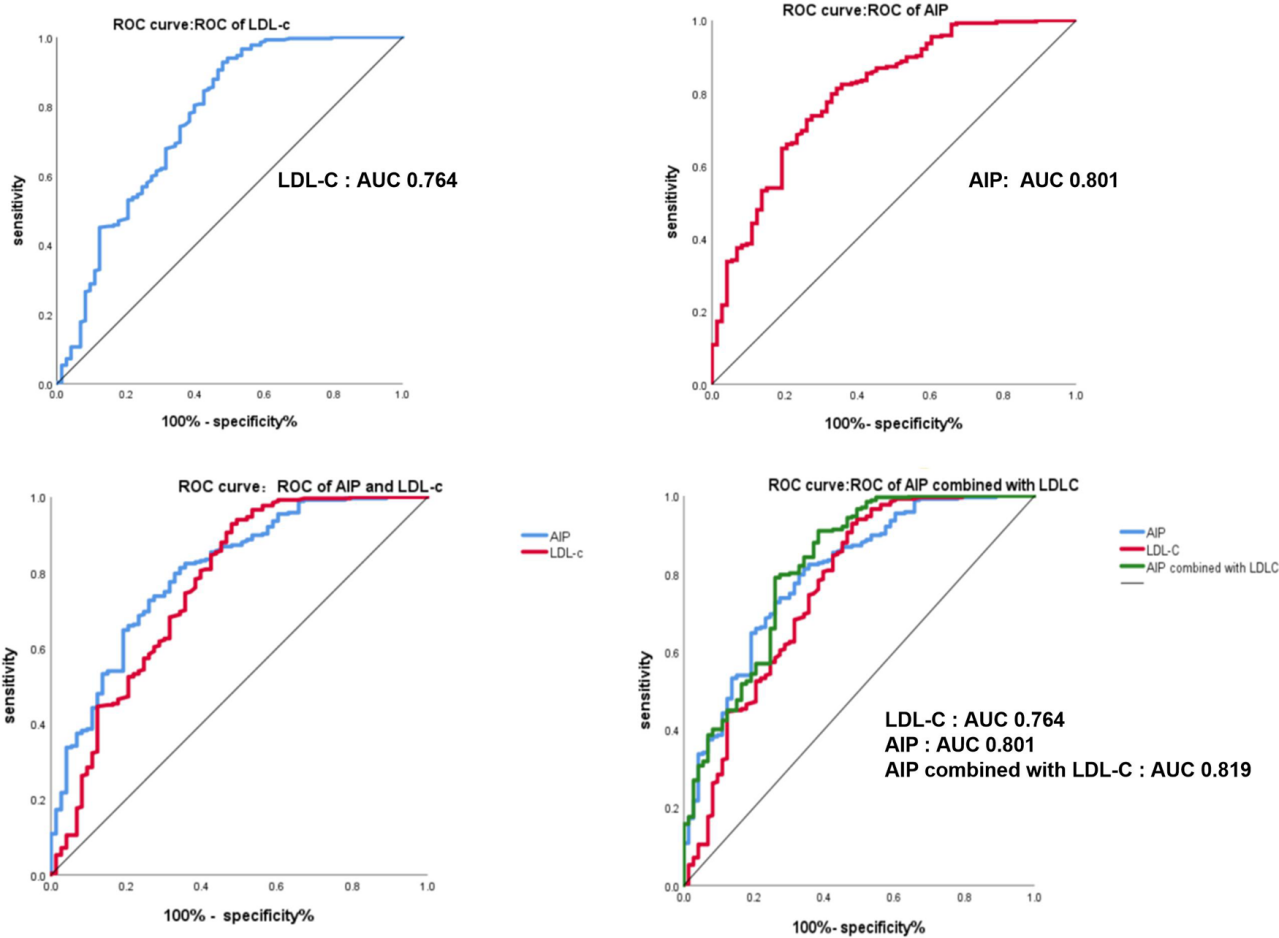
The ROC curve of AIP, LDL-C and AIP combined with LDL-C in predicting th ability of acute myocardial infarction.

After adjusting for confounding factors such as smoking, history of diabetes, systolic blood pressure, and diastolic blood pressure at admission, the ability of AIP, LDL-C, and the combination of AIP and LDL-C to predict AMI was assessed using ROC curves. The results indicated that AIP had a best cut-off value of −0.06142, with a sensitivity of 81.3%, specificity of 65.8%, and an AUC of 0.801 (95% CI: 0.743–0.859, *P *< 0.001). The optimal cut-off value of LDL-C for predicting AMI was 1.84, with a sensitivity of 0.929, specificity of 52.1%, and an AUC of 76.4% (95% CI: 0.693–0.834, *P *< 0.001). For the combination of AIP and LDL-C, the optimal cut-off value was 0.76, with a sensitivity of 79%, specificity of 74%, and an AUC of 0.819 (95% CI: 0.759–0.879, *P *< 0.001) (Figure 3, Table 3). ROC curve analysis was utilized to assess the predictive ability of AIP and the combination of AIP and LDL-C in identifying acute myocardial infarction caused by occlusion in different coronary arteries, namely left anterior descending (LAD), left circumflex (LCX), and right coronary artery (RCA) (Table 4). The findings of subgroup analysis based on AIP alone demonstrated the following results: for LAD, the optimal cut-off value was −0.0513, with a sensitivity of 79.2%, specificity of 67.1%, and an AUC of 0.787 (95% CI: 0.721–0.854, *P *< 0.001); for LCX, the optimal cut-off value was −0.0666, with a sensitivity of 88.5%, specificity of 45.2%, and an AUC of 0.673 (95% CI: 0.583–0.764, *P *= 0.001); and for RCA, the optimal cut-off value was 0.014, with a sensitivity of 79%, specificity of 74%, and an AUC of 0.834 (95% CI: 0.771–0.897, *P *< 0.001). Furthermore, the results of subgroup analysis based on AIP combined with LDL-C revealed the following outcomes: for LAD, the optimal cut-off value was 0.577, with a sensitivity of 80.8%, specificity of 72.6%, and an AUC of 0.819 (95% CI: 0.756–0.883, *P *< 0.001); for LCX, the optimal cut-off value was 0.379, with a sensitivity of 83.6%, specificity of 67.1%, and an AUC of 0.820 (95% CI: 0.751–0.888, *P *< 0.001); and for RCA, the optimal cut-off value was 0.40, with a sensitivity of 86.4%, specificity of 68.5%, and an AUC of 0.836 (95% CI: 0.773–0.898, *P *< 0.001) (Table 4).

**Table 3:** The ROC curve related indicators of AIP, LDL-C and AIP combined with LDL-C in predicting the ability of acute myocardial infarction.

Variable	AUC (95% CI)	The best cut-off value	Sensitivity	Specificity	*P*
AIP	0.801 (0.743,0.859)	−0.06142	81.3%	65.8%	<0.001*
LDL-C	0.764 (0.693,0.834)	1.84	92.9%	52.1%	<0.001*
AIP combined with LDL-C	0.819 (0.759,0.879)	0.756107	79%	74%	<0.001*

(LDL-C), low-density lipoprotein cholesterol; (AIP), Atherogenic index of plasma; (95% CI), 95%; (ROC) curves, confidence intervalReceiver operating characteristic.

* *P* < 0.05.

**Table 4:** The ROC curve related indicators of AIP and AIP combined with LDL-C in predicting the ability of AMI due to LAD, LCX and RCA blockage.

Variable (Blocked coronary arteries)	AUC (95% CI)	The best cut-off value	Sensitivity	Specificity	*P*
AIP (LAD)	0.787 (0.721,0.854)	−0.0513	0.792	0.671	<0.001*
AIP (LCX)	0.673 (0.583,0.764)	−0.0666	0.885	0.452	0.001*
AIP (RCA)	0.834 (0.771,0.897)	0.014	0.79	0.74	<0.001*
AIP combined with LDL-C (LAD)	0.819 (0.756,0.883)	0.576635	0.808	0.726	<0.001*
AIP combined with LDL-C (LCX)	0.820 (0.751,0.888)	0.3792761	0.836	0.671	<0.001*
AIP combined with LDL-C (RCA)	0.836 (0.773,0.898)	0.3992453	0.864	0.685	<0.001*

(LAD), left anterior descending coronary artery;(LCX), left circumflex coronary artery; (RCA), right coronary artery; (LDL-C), low-density lipoprotein cholesterol; (AIP), Atherogenic index of plasma; (95% CI), 95% confidence interval; (ROC) curves, Receiver operating characteristic.

* *P* < 0.05.

## 4. Discussion

The causes of AMI include vascular stenosis, myocardial ischemia, hypoxia, and myocardial injury caused by coronary atherosclerosis, therefore it is crucial to actively control atherosclerosis. Abnormal lipid metabolism plays a critical role in the progression of coronary atherosclerosis, calcified plaque formation, and deterioration (13, 14). Prior studies have shown that both high TG levels and low HDL-C levels are significant markers of CVD (15).

The AIP is the logarithm of the ratio of TG and HDL-C concentration, which indirectly reflects the size of sdLDL-C particles. Compared to LDL-C, sdLDL is more likely to invade and deposit on the arterial and is easily oxidized to oxidized LDL to accelerate the process of atherosclerosis (16). There is ample evidence that sdLDL-C has a greater atherogenic potential than other LDL subfractions and that the sdLDL-C ratio is a better predictor of cardiovascular disease than LDL-C (17). The sdLDL-C has a strong atherosclerotic effect, primarily due to its small particle diameter and a strong affinity with proteoglycans in the intima of the artery. It is easily modified by oxidation, has a low affinity with receptors, slow clearance, and long retention time. Therefore, the new lipid parameter AIP reflects subtle interactions in lipid metabolism and can serve as a better index for assessing cardiovascular risk. This study investigated the relationship between AIP and patients with AMI to evaluate the correlation between the two. The main finding of the study is that the AIP index is an independent risk factor for AMI, which is a major cause of high morbidity, hospitalization, and mortality worldwide. Utilizing the AIP index alone, as well as in conjunction with LDL-C, can serve as effective predictors of AMI.

Previous research has indicated that AIP may be a useful indicator for predicting the risk of rapid progression of coronary atherosclerosis (11), as well as for diagnosing and predicting the prognosis of coronary artery disease (CAD) in certain populations, such as the elderly and postmenopausal women (16, 18–21). Meta-analysis suggests that AIP may be an independent risk factor for CAD (22). Additionally, some studies have suggested that AIP is associated with the prognosis of CHD patients after PCI, regardless of whether they have diabetes (23–25). However, while there have been numerous studies examining the association between AIP and CHD, few have investigated its diagnostic value specifically for AMI. This study sought to assess the diagnostic potential of AIP for AMI and to compare its diagnostic efficacy with that of LDL-C, a traditional risk factor, and the combined diagnostic predictive value of the two.

This study found that AIP had a high predictive value for AMI, and when AIP was combined with LDL-C, the predictive power of AIP for AMI could be increased. Furthermore, prior research has not examined the variability in the predictive capability of AIP in patients with diverse types of coronary artery occlusion. Therefore, in this study, subgroup analysis was performed to assess the predictive value of AIP and AIP combined with LDL-C for myocardial infarction attributed to different types of coronary artery occlusion. The findings revealed that when AIP was utilized as a standalone marker, its predictive value for LCX occlusion was limited. However, when AIP was combined with LDL-C, the predictive value was similar across all three groups.

Since AIP can indirectly reflect sdLDL-C, we postulate that the impact of sdLDL-C may exhibit divergent outcomes in distinct coronary artery pairs, as variances in anatomical and hemodynamic features of coronary arteries may influence the deposition of sdLDL-C and the formation of plaques within the arterial wall. These discrepancies may be attributed to variations in the branching pattern of coronary arteries, the extent of blood supply territory, and differences in blood flow velocity and vessel wall characteristics among different arteries. For instance, the LAD is commonly associated with a high incidence of coronary artery lesions, whereas the RCA and LCX are typically less affected. Further investigation with larger sample sizes may be warranted to elucidate the underlying reasons.

Therefore, our study highlights that AIP is a new independent risk factor for AMI, and both AIP and LDL-C can be used in clinical settings to predict the occurrence of AMI. Among them, AIP may be more meaningful for the prediction of AMI caused by LAD and RCA obstruction. AIP has the potential to become a reliable, easily accessible, and low-cost diagnostic index in remote areas where CAG is not readily available. Furthermore, previous studies have shown that early intervention of AMI is extremely important for improving the prognosis of patients, and there is no significant difference in the prognosis of patients with AMI treated with thrombolysis and PCI within 3 h. AIP can be used to determine the likelihood of AMI when the markers of myocardial injury are not elevated in the early stages of AMI or when chest pain is mild or has an atypical location, guiding further thrombolysis or PCI therapy.

In conclusion, our findings suggest that the AIP index is an independent risk factor for AMI, and both the AIP index alone and in combination with LDL-C can serve as predictive tools for AMI.

## 5. Limitation

However, several limitations in this study should be acknowledged. Firstly, the study design is retrospective and the sample size is small, consisting of only hospital patients, which introduces potential selection bias. Secondly, the sample size in this study is inadequate, and the study was conducted in a single region in China, which may limit the generalizability of the findings. Therefore, future studies with larger sample sizes and broader geographic representation are needed. Thirdly, the diagnostic accuracy of AIP should be compared with commonly used markers for AMI, such as troponin. Therefore, multicenter prospective studies with larger sample sizes are necessary to validate the predictive value of AIP and AIP combined with LDL-C for AMI, and to compare them with established clinical indicators for myocardial infarction, such as troponin levels and electrocardiogram changes.

## Data Availability

The raw data supporting the conclusions of this article will be made available by the authors, without undue reservation.
